# Treatment of three-level cervical spondylotic myelopathy using ACDF or a combination of ACDF and ACCF

**DOI:** 10.3389/fsurg.2022.1021643

**Published:** 2022-09-16

**Authors:** Xiaoming Tian, Hongwei Zhao, Felicity Y. Han, Samuel Rudd, Zhaohui Li, Wenyuan Ding, Sidong Yang

**Affiliations:** ^1^ Department of Spinal Surgery, The Third Hospital of Hebei Medical University, Shijiazhuang, China; ^2^ The Third Hospital of Hebei Medical University, Shijiazhuang, China; ^3^ Australian Institute for Bioengineering and Nanotechnology, The University of Queensland, Brisbane, Australia; ^4^ School of Chemical Engineering, The University of Queensland, Brisbane, Australia

**Keywords:** ACDF, ACCF, cervical spondylotic myelopathy, anterior decompression, fusion, anterior column height

## Abstract

**Objectives:**

This study aims to compare the outcomes between two anterior decompression and fusion techniques to treat multilevel cervical spondylotic myelopathy (MCSM).

**Methods:**

After the screening for eligibility, a total of 66 patients were admitted to this study. These participants underwent anterior surgeries due to MCSM in our hospital between June 2016 and July 2018. All participants underwent either the anterior cervical discectomy and fusion (ACDF) surgery (ACDF group) or the combination of ACDF and anterior cervical corpectomy and fusion (ACCF), which was the anterior cervical hybrid decompression and fusion (ACHDF) surgery group. All the patients were followed up ≥18 months, the average latest followed up time was 23.64 (±2.69) months. The length of hospitalization, operation time, blood loss, visual analog scale (VAS), Japanese Orthopaedic Association (JOA) score, improvement rate, Hounsfield units (HU) of C3–C7, cobb angle, and anterior column height of fusion levels pre and post operation were analyzed.

**Results:**

There were no statistical differences between the ACDF and ACHDF groups regarding the length of hospitalization, operation time, blood loss, HU of C3–C7, VAS, JOA score, improvement rate, cobb angle, and anterior column height in fusion levels in pre-operation and 3 months after operation (all *P* > 0.05). However, compared with the ACHDF group, the ACDF group achieved significantly better improvement in the anterior column height of fusion levels in the final 18–29 months post-operatively (*P* < 0.05).

**Conclusions:**

Both approaches of ACDF alone and a combination of ACDF and ACCF can achieve satisfactory outcomes in the treatment of MCSM, but ACDF has better outcomes in maintaining anterior column height of fusion levels.

## Introduction

Cervical spondylotic myelopathy (CSM) is the most common cause of spinal cord dysfunction (1). CSM often presents with clinical symptoms and signs of impaired upper motor neurons. Multilevel cervical spondylotic myelopathy (MCSM) is a pathological change that affects three or more segments in the cervical intervertebral disc and the surrounding tissues. MCSM often goes with hyperosteogeny, facet joint degeneration or hypertrophy, and ossification of the peripheral ligament (2). It may reduce patients’ ability to do daily activities or even lead to paralysis, which not only reduces patients’ quality of life, but also causes a substantial social-economic burden. The outcome of conservative treatment is usually insufficient to treat this condition. Immediate surgical intervention is always required once MCSM is diagnosed.

MCSM is generally caused by pathologies that directly compress the spinal cord on the ventral side of the spinal column. Clinically, there are several surgical procedures used to treat MCSM, with two basic approaches (3). The first is the anterior approach, which aims to directly relieve the compression, including anterior cervical discectomy with fusion (ACDF), anterior cervical corpectomy and fusion (ACCF), anterior cervical hybrid decompression and fusion surgery (ACHDF, the combination of ACDF and ACCF), anterior approach with zero-profile devices and artificial disc replacement (ADR). The second one is to widen the spinal canal indirectly by using the bowstring effect *via* a posterior approach including laminoplasty and laminotomy. Due to the pathologies of MCSM, the anterior approach is an effective but less invasive surgical procedure for patients whose compression is less severe. With the advantages of techniques and the popularization of surgical approaches, anterior surgery is becoming increasingly common for treating MCSM.

Among various techniques in the anterior approach, the zero-profile devices and ADR are newly developing devices which are not widely used for MCSM due to the relatively high surgical skill requirement of these devices and a narrow application range (4–7). Multiple segmental ACCF greatly changes the cervical spine structure and causes massive injuries (8). Studies have shown that ACCF in MCSM has no significant advantages over other procedures in terms of surgical outcomes (9–11). Currently, the two main anterior procedures used to treat MCSM are multi-segmental ACDF and the combination of ACDF and ACCF. These two approaches have demonstrated similar effectiveness and safety (8). However, there have been few comparisons between these two procedures as to which one delivers better outcomes for patients. Thus, the current study aimed to compare ACDF alone with the combination of ACDF and ACCF in treating three-level MCSM, with the purpose of determining the best procedure.

## Patients and methods

### Data collection

The patients who underwent anterior surgeries for MCSM with intervertebral disc herniation in our hospital between June 2016 and July 2018 were reviewed in our study. This research was approved by the Ethics Committee of the Third Hospital of Hebei Medical University; all patients agreed to participate in this study for publishing of data and images. Inclusion criteria were as follows: (1) the imagelogical examination showed three or more levels of compression; (2) fatigue or pain in the neck and shoulder, upper limb numbness, loss of muscle tone, or other symptoms caused by peripheral nerve injury in context of excluding other systemic diseases; (3) hypertonia, hyperreflexia, positive pathological; signs or symptoms of upper motor neuron injury. The exclusion criteria were as follows: (1) diagnosed with multi-segmental cervical spondylotic radiculopathy; (2) cervical surgery history; (3) cervical vertebral fracture, spinal cord injury; (4) cervical tumor, inflammation; (5) serious ossification of the posterior longitudinal ligament. To ensure the patients’ maximum benefits, the patients with severe compression to the spinal cord which is difficult to decompress using the ACDF surgery, and those patients with the compression came from the posterior vertebral body, were chosen to perform ACDF and ACCF combined surgery.

### Surgical procedures

**Figure 1:**
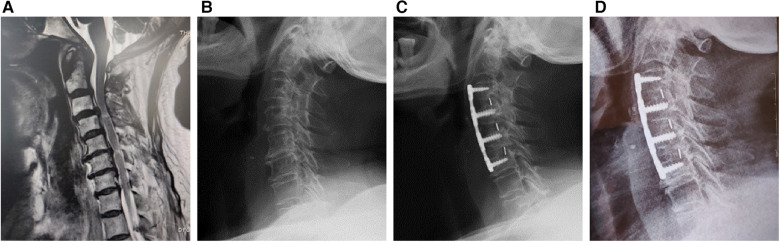
Male, 69-year-old, underwent three-level anterior cervical discectomy and fusion (ACDF) surgery. (**A**) The pre-operational magnetic resonance imaging (MRI), (**B**) the x-ray of pre-operation, (**C**) operation after 3 months, and (**D**) the final follow-up.

**Figure 2:**
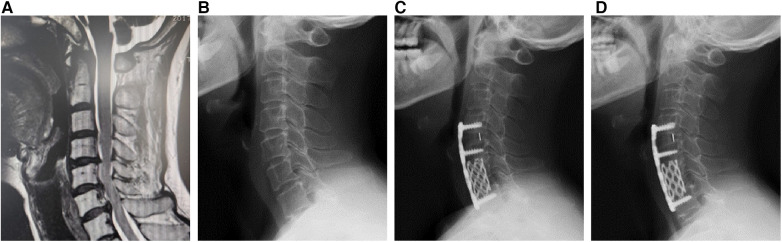
Female, 50-year-old, underwent one-level anterior cervical discectomy and fusion (ACDF) and one-level anterior cervical corpectomy and fusion (ACCF) surgery. (**A**) The pre-operational MRI, (**B**) the x-ray of pre-operation, (**C**) operation after 3 months, and (**D**) the final follow-up.

The operation level was determined by medical history, physical examination, and radiological examination. Before the operation, all the patients underwent tracheoesophageal push training to prevent post-operation sputum and dysphagia. Under general anesthesia, a Smith-Robinson incision was made on the right side of the neck. In the ACDF group (Figure 1), after the discectomy, the suitable poly ether ether ketone (PEEK) cages were implanted. In the ACHDF group (Figure 2), the severe compression levels were followed by vertebral corpectomy, and titanium mesh cages (TMC) were implanted with autogenous bone. Then, a single-level ACDF was implemented on the adjacent level. Both groups were fixed by a titanium plate with screws (eight in the ACDF surgery and six in the ACHDF surgery) that fit the centrum. All the surgeries were performed by the same surgeon.

### Radiological parameters

**Figure 3:**
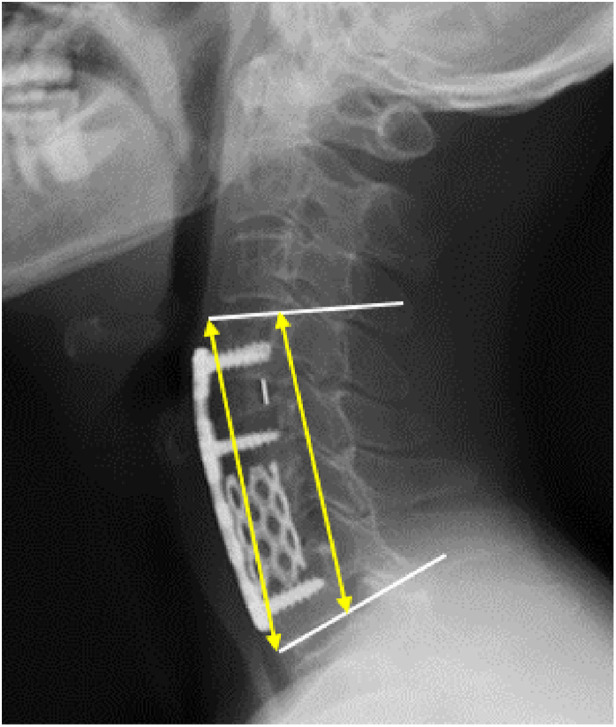
The height of the anterior column (the mean value of two yellow arrows) and the cobb angle of fusion segments (the angle of two white lines).

**Figure 4:**
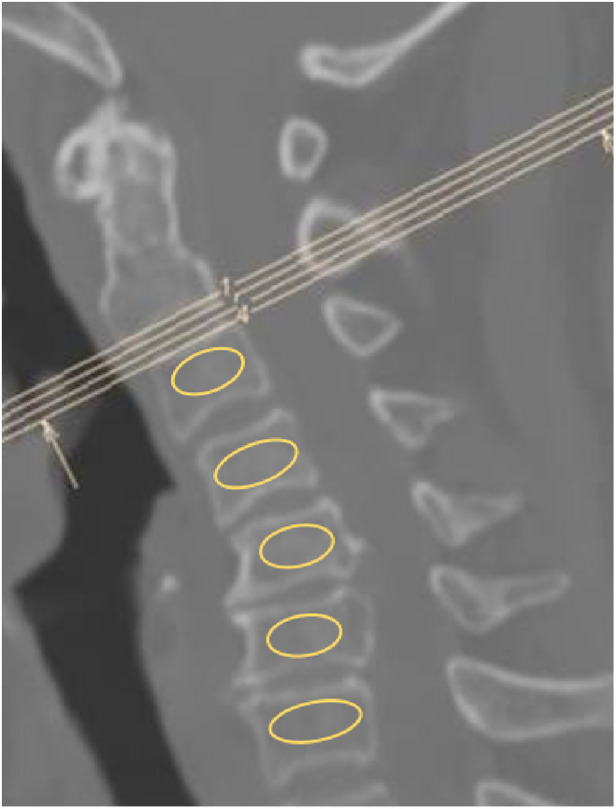
Using an elliptical region of interest function to evaluate the hounsfield units (HU) value in median sagittal computed tomography (CT) scan of the cervical spine, select the largest possible range of cancellous bone without including cortical bone.

The radiological parameters were the cobb angle of fusion segments, the height of the anterior column in sagittal x-ray, and Hounsfield units (HU) values in computed tomography (CT). All data were measured by two researchers and the average value of two measurements was analyzed. Another expert was asked to evaluate the data to ensure accuracy. The cobb angle of fusion segments was measured as the angle between the upper endplate of the upper fusion vertebrae and the lower endplate of the lower fusion vertebrae (12). The height of the anterior column was measured as the average value of the anterior-inferior intersection of the lower vertebral body and the anterior-superior intersection of the upper vertebral body (Figure 3). The HU values (13) were measured using an elliptical region of interest function in the median sagittal position of the cervical spine (Figure 4).

### Clinical assessment

Clinical parameters include the Japanese Orthopaedic Association (JOA) and visual analog scale (VAS), length of hospitalization, operation time, blood loss and improvement rate. All the patients underwent preoperative evaluation, and were followed up for 23.64 (±2.69) months on average.

### Statistical analysis

All data were analyzed by SPSS 21.0 (IBM, Armonk, New York, United States) software. Continuous variables are presented as mean ± standard deviation (SD) when normally distributed, and as median (interquartile ranges, IQR) when the distribution was skewed. Independent *t-*tests were performed to compare radiological and clinical parameters for independent samples. Chi-square test was performed for categorical data. For continuous variables but not normally distributed, Mann-Whitney U-test was applied. Repeated measure ANOVA and the generalized estimating equation were used to compare the repeated measurement data. *P* < 0.05 was considered statistically significant.

## Results

A total of 66 patients were enrolled in this study, and none of these patients experienced any severe complications. The general data between the ACDF and ACHDF groups did not show statistical differences in gender, age, BMI, complications, tobacco and alcohol addiction, course of the disease, and operation sections (Table 1). The HU values of cervical vertebrae C3–C7 were used to evaluate the bone mineral density and resistance to external forces in the two groups. In the ACDF group, the HU values were 321.91, 311.48, 310.83, 276.20, and 246.96 from C3 to C7 respectively. While in the ACHDF group the HU values were 337.93, 320.69, 320.80, 271.58 and 262.93 from C3 to C7 respectively (Table 2). In each vertebral segment, there were no statistical differences regarding to the HU values between the ACDF and ACHDF groups (*P* > 0.05).

**Table 1:** Comparison of general data between the two groups of ACDF and ACHDF.

	ACDF (*n* = 43)	ACHDF (*n* = 23)	*Z* value	*P* value
Gender (female/male)	16/27	12/11	–	0.241
Age (year)	56.4 (±9.62)	57.3 (± 9.21)	–	0.712
BMI	25.35 (IQR = 3.60)	25.26 (IQR = 2.93)	−0.040	0.968
Diabetes	4	6	–	0.070
Hypertension	12	10	–	0.201
Smoking	2	3	–	0.220
Drinking	6	4	–	0.711
Course of the disease (month)	3 (IQR = 10.84)	7 (IQR = 45.00)	−1.647	0.099
Operation sections				0.534
C3–C6	20	8	–	
C4–C7	20	14	–	
C3–C4/C5–C7	3	1	–	

ACDF, the anterior cervical discectomy and fusion surgery; ACHDF, the anterior cervical hybrid decompression and fusion surgery (the combination of ACDF and ACCF); BMI, body mass index; IQR, interquartile ranges.

**Table 2:** The comparison of HU in C3–C7 of the two groups of ACDF and ACHDF.

	ACDF (*n* = 43)	ACHDF (*n* = 23)	*Z* value	*P* value
C3	321.91 (IQR = 96.92)	337.93 (IQR = 109.93)	−0.828	0.408
C4	311.48 (IQR = 103.48)	320.69 (IQR = 112.73)	−0.357	0.721
C5	310.83 (IQR = 69.13)	320.80 (IQR = 104.40)	−1.204	0.228
C6	276.20 (IQR = 104.29)	271.58 (IQR = 108.02)	−0.101	0.920
C7	246.96 (IQR = 78.33)	262.93 (IQR = 112.81)	−1.151	0.250

ACDF, the anterior cervical discectomy and fusion surgery; ACHDF, the anterior cervical hybrid decompression and fusion surgery (the combination of ACDF and ACCF); HU, Hunsfield units; IQR, interquartile ranges.

Comparisons of the length of hospitalization, operation time, blood loss, VAS, JOA score, and improvement rate showed no statistical differences between the ACDF and ACHDF groups (*P* > 0.05). In both ACDF group and ACHDF group, post-operation VAS and JOA scores showed improvements compared to pre-operative scores. The median VAS score decreased from 2 to 1 in the ACDF group, and from 3 to 1 in the ACHDF group. The JOA score of the ACDF group increased from 8 to 14, while in the ACHDF group increased from 8 to 13 (Table 3).

**Table 3:** The comparison of length of hospitalization (days), operation time (min), blood loss (ml), VAS, JOA score and improvement rate (%) of the two groups of ACDF and ACHDF.

	ACDF (*n* = 43)	ACHDF (*n* = 23)	*Z* value	*P* value
Hospitalization (days)	12.11 (±4.02)	13.65 (±3.27)	–	0.121
Operation time (min)	133.63 (±34.22)	136.09 (±41.40)	–	0.797
Blood loss (ml)	200 (IQR = 200)	200 (IQR = 200)	−0.314	0.754
VAS (pre-operation)	2 (IQR = 4)	3 (IQR = 3)	−0.979	0.328
VAS (last follow-up)	1 (IQR = 2)*	1 (IQR = 2)*	−0.170	0.865
JOA (pre-operation)	8 (IQR = 2)	8 (IQR = 2)	−0.868	0.385
JOA (last follow-up)	14 (IQR = 1)*	13 (IQR = 2)*	−1.749	0.080
Improvement rate (%)	62.50 (IQR = 14.44)	50.00 (IQR = 25.56)	−1.619	0.105

ACDF, the anterior cervical discectomy and fusion surgery; ACHDF, the anterior cervical hybrid decompression and fusion surgery (the combination of ACDF and ACCF); VAS, visual analog scale; JOA, Japanese orthopaedic association; IQR, interquartile ranges.

*Means statistically significant between pre-operation and last follow-up in the same group.

The anterior column height in the ACDF group was 76.96 (±9.72) mm, 80.89 (±9.26) mm, and 79.85 (±9.20) mm pre-operation, 3 months after surgery, and the last follow-up respectively. In the ACHDF group, the anterior column height was 73.10 (±8.62) mm, 76.56 (±7.30) mm, and 75.27 (±7.41) mm pre-operation, 3 months after surgery, and the last follow-up, respectively. The anterior column height at the final follow-up was lower compared to 3 months after surgery in both groups. However, there was a significant improvement when compared to the pre-operation (*P* < 0.05). In the last follow-up, the anterior column height was significantly higher in the ACDF than in the ACHDF groups (*P* < 0.05), indicating that the ACDF group was better than the ACHDF group. Although the improvement of the cobb angle showed statistical differences between the last follow-up and pre-operation within the ACDF group and not in the ACHDF group, there was no statistical difference between the ACDF and ACHDF groups (Table 4).

**Table 4:** The comparison of cobb angle (degree) and anterior column height (mm) of the two groups of ACDF and ACHDF.

	ACDF (*n* = 43)	ACHDF (*n* = 23)	*P* value
Cobb (pre-operation)	8.67 ± 9.54	10.09 ± 10.86	0.587
Cobb (3 months)	12.53 ± 5.95**	12.87 ± 6.92**	0.838
Cobb (last follow-up)	11.58 ± 5.89*^,^***	11.48 ± 6.73***	0.949
Height (pre-operation)	76.96 ± 9.72	73.10 ± 8.62	0.116
Height (3 months)	80.89 ± 9.26**	76.56 ± 7.30**	0.057
Height (last follow-up)	79.85 ± 9.20*^,^***	75.27 ± 7.41*^,^***	*0*.*044*

ACDF, the anterior cervical discectomy and fusion surgery; ACHDF, the anterior cervical hybrid decompression and fusion surgery (the combination of ACDF and ACCF).

*Means statistically significant between pre-operation and last follow-up in the same group.

**Means statistically significant between pre-operation and 3-month follow-up in the same group.

***Means statistically significant between the 3-month follow-up and last follow-up in the same group.

### Discussion

#### The surgical methods for MCSM

MCSM is a multi-factor caused disease, including intervertebral disc degeneration, narrowing of the disc space, and osteophyte formation that changes the curvature of the cervical spine to be straight or reverse. The nerve damage is progressive and can cause disability. Conservative treatment is generally ineffective, and immediate surgical intervention is required (14). Many surgical procedures are used, including anterior, posterior, and combined anterior-posterior approaches (15). The anterior approach includes ACDF, ACCF, the combination of ACDF and ACCF, and with the use of zero-profile devices and ADR. The posterior approach includes laminectomy with or without fusion and laminoplasty (16). The combined anterior-posterior approaches include the first or second stage surgery and is only used to provide a greater effect on deformity correction. Due to the higher mortality and morbidity rates (17), the combined anterior-posterior surgery is not preferred by surgeons. Our previous study has shown that the combined anterior-posterior with posterior instrumented fixation is a good choice to treat adjacent segmental disease caused by ACCF (18).

The anterior approach was proposed by Smith and Robinson in 1958 (19) and was acknowledged by spinal surgeons. The anterior approach can remove the compression by excising the herniated disc, the osteophyte behind the vertebrae, and the posterior longitudinal ligament, especially in single-level cervical spondylosis (20). To date, the consensus on the best approach to treat MCSM has not been achieved due to the complex pathogenesis and compression from the front and rear of the cervical vertebrae. For patients with compression from the front, anterior approach surgeries are usually selected, including ACCF, ACDF, and the combination of ACDF and ACCF.

Long-segments ACCF is not the first choice usually. The direct vision is available using ACCF, with a large operative field and thorough decompression, but the damage and change to the anterior and middle columns are large, which cannot be ignored. In addition, multilevel segment fixation without enough bone structure induces more stress that may lead to screws loosening, displacement and other postoperative complications (21).

A multilevel ACDF surgery can alleviate the compression by removing the disc, osteophyte, and posterior longitudinal ligament directly. The surgery retains the structural stability of columns and restores physiological curvature. Multilevel ACDF also fits skipped-level cervical spondylosis patients to protect the normal disc (22), and is even chosen for cervical kyphosis. Moreover, ACDF is clinically favored due to a minimal barrier to entry and short learning curve for trainee surgeons. However, the ACDF approach also has certain drawbacks, such as tunnel vision, a limited operational field, and the inability to alleviate compression below the targeted disc level.

The combination of ACDF and ACCF is a technique that combines one segment of ACDF with ACCF to maximize the benefits of the two surgical methods. With a broader view of the severe segments and less damage to the mild segments, ACCF releases compression that comes from the vertebral bodies while ACDF removes moderate compression that comes from the diseased disc. However, our data show that ACDF has better outcomes in maintaining anterior column height in fusion levels when compared with a combination of ACDF and ACCF (Table 4).

### The effect of restoring the anterior column height and curvature on patient outcomes

Upon imaging, MCSM frequently exhibits a reduction in disc height, which indicates compression and narrowing of the nerve root canal. Loss of anterior column height can cause folds in the posterior longitudinal ligament and ligamentum flavum, squeezing the spinal cord. If the height loss cannot be restored during surgery, the volume of the spinal canal will not be regained. Therefore, it is necessary to gain height and regain the curvature during the surgery to obtain satisfactory outcomes (23). Aiming to enlarge the nerve root canal and restore the tension of surrounding tissues, reconstruction of the anterior column with bone grafting can effectively remove compressive factors and immediately increase the anterior column height.

In our study, both multilevel ACDF and the combination of ACDF and ACCF can increase the anterior column height and improve VAS and JOA scores (Table 3). The height of the ACDF and ACHDF groups was lower in the final follow-up than in the 3-month follow-up, but without statistical difference. This result possibly relates to an adaptive response to the implant which can cause a small amount of subsidence of the anterior cervical column (24). Additionally, our study showed that the anterior column height in the ACHDF group was significantly lower compared to the ACDF group in the final follow-up. Also, the HU values did not show statistical differences between the ACDF and ACHDF groups. Previous reports showed that HU values were associated with compressive tolerance and represent bone mineral density in detecting the degree of osteoporosis (25), meaning that osteoporosis in the ACDF and ACHDF groups can be ignored. The difference in column height is most likely due to TMC compressing cancellous bone more firmly over the entire vertebral body than in the interbody fusion cage (26). According to other studies, a vertical reduction of more than 3 mm in the intervertebral disc space is related to severe narrowing of the neuroforamen (27). There is a risk of secondary surgical revision if the continuous subsidence and loss of curvature lead to a secondary compression to the nerve roots and spinal cord, while some studies showed that there is no relationship between subsidence and clinical outcomes (28). Despite the fact that three patients experienced screw issues, it has been believed that inadequate bone fusion causes implant problems rather than subsidence (12). The preservation of the endplate, the degree of osteoporosis, and the length of the implantation materials can impact the patient’s prognosis by influencing the column height at the fusion segments (29).

The restoration of cervical curvature is an important indicator of the efficacy of anterior cervical spine surgery. The maintenance of cervical spine curvature is a critical factor in preventing the deterioration of neurological function (23, 30). Most healthy cobb angle of C2–C7 ranges from 20° to 25°. This physiological pronation angle has a cushioning effect on the spinal cord. MCSM often causes the straightening or even reversal of the cervical spine. These changes would further aggravate the degeneration of the adjacent discs, small joints, and tissues. Studies on the vascular supply to the spinal cord have found that the decreased anterior-posterior diameter of the spinal cord is strongly correlated with the decreased spinal cord blood volume, and spinal cord ischemia induces neural function disorders (31). Axial symptoms also occur in patients with reverse cervical curvature. In the current study, the cobb angle was improved in both ACDF and ACHDF groups compared with the pre-operation. In the ACHDF group, the cobb angle showed no statistical differences compared with pre-operation. This result may be due to the difference of subsidence. The subsidence of the anterior intervertebral height was more than that of the posterior intervertebral height when ACCF was performed with the TMC (12). However, another study showed the opposite results (28). We speculate that it might be associated with the immediate cervical curvature of the patients when the fusion device is implanted. In addition, the depth of implant insertion, the degree of fit between the implant and the endplate, and the potential influence of the ACDF segment on the ACCF segment remain controversial and need to be further investigated.

It is worth noting that this is a single-center retrospective study. Due to the relatively small number of severe MCSM cases, the sample size is small, and the number of patients in the ACDF and ACHDF groups is unbalanced. Therefore, a multi-center prospective study is expected to further confirm our findings. Additionally, because of the short follow-up period, the exact timing of when the differences in anterior column height occurred is unknown. Therefore, further investigation of the maintenance of cobb angle and the height over a long period is required.

## Conclusion

ACDF alone and the combination of ACDF and ACCF procedures have similar treatment outcomes in the treatment of MCSM. Compared with the combination of ACDF and ACCF procedures, ACDF alone can better maintain anterior column height.

## Data Availability

The raw data supporting the conclusions of this article will be made available by the authors, without undue reservation.
